# Supplementary material for: The effects of high-frequency repetitive transcranial magnetic stimulation on negative symptoms in schizophrenia patients: A systemic review and meta-analysis
Source: PLoS One. 2025 Dec 11;20(12):e0337847. doi: 10.1371/journal.pone.0337847 (PMC12697975; doi:10.1371/journal.pone.0337847)
Supplement: S1 Checklist — (DOCX) [file pone.0337847.s001.docx]

| **Section and Topic** | **Item #** | **Checklist item** | **Location where item is reported** |
| --- | --- | --- | --- |
| **TITLE** | | |  |
| Title | 1 | The effects of high-frequency repetitive transcranial magnetic stimulation on negative symptoms in schizophrenia patients: A systemic review and meta-analysis | Page 1 |
| **ABSTRACT** | | |  |
| Abstract | 2 | Objective: The research on high-frequency repetitive transcranial magnetic stimulation (rTMS) is very little, and its effectiveness have hardly been provided conclusive evidence. This systematic review and meta-analysis aimed to determine the effects of high-frequency repetitive transcranial magnetic stimulation on negative symptoms in schizophrenia patients.  Methods: Six databases (PubMed, Embase, Web of Science, SinoMed, PsycINFO and MEDLINE) were searched from inception to March 2024. Relevant data were extracted and analysed by two independent investigators using Cochrane RevMan software (version 5.3) and R software (version 4.3.1).  Results: A total of 17 RCTs were included in the current meta-analysis. Compared with sham treatment, active rTMS showed advantage in treating negative symptoms of schizophrenia [standardized mean difference (SMD): -0.22, 95% confidence interval (CI): -0.38, -0.05, P = 0.009; I2 = 9%]. Subgroup analysis revealed that rTMS with a total treatment course of more than 15 sessions [SMD: -0.31 (95% CI: -0.49, -0.13), P = 0.0007; I2 = 0%], active rTMS with a frequency of 20 Hz [SMD: -0.40 (95% CI: -0.67, -0.13), P = 0.004; I2 = 0%], and active rTMS targeting the dorsolateral prefrontal cortex (DLPFC) [SMD: -0.25 (95% CI: -0.40, -0.09), P = 0.003; I2 = 0%] were significantly effective in terms of reducing negative symptoms.  Conclusion: The effect of rTMS on negative symptoms is influenced by rTMS parameters. We recommend the rTMS located in the DLPFC, with a frequency higher than 20 Hz, or more than 15 sessions of stimulation to reduce negative symptoms of schizophrenia. | Page 1-2 |
| **INTRODUCTION** | | |  |
| Rationale | 3 | To reduce the negative symptoms are one of the challenges of schizophrenia. Recent clinical studies have suggested that up to 60% of patients may exhibit prominent negative symptoms that necessitate treatment. However, antipsychotic treatments, such as antipsychotic drugs with dopamine D2 antagonists or partial D2 agonists, are not optimal method. While these drugs may be effective for treating some positive symptoms, they often fail to address negative symptoms and can lead to side effects such as weight gain and metabolic syndrome. Furthermore, the clinical diagnosis of negative symptoms is challenging because schizophrenia patients may not recognize the impact of negative symptoms and are unlikely to report them. | Page 3 |
| Objectives | 4 | In recent years, several meta-analyses and reviews have also provided evidence supporting the use of high-frequency rTMS for treating these symptoms. However, the frequency, location and sessions of high-frequency rTMS remain controversial. Recently, high-frequency rTMS also has therapeutic implications for depression, but studies have suggested that rTMS may not have a significant benefit on positive symptoms of schizophrenia. Therefore, we conducted this systematic review and meta-analysis to assess the effectiveness of high-frequency rTMS for reducing negative symptoms among schizophrenia patients, and to assess the effectiveness for depression and positive symptoms of schizophrenia. | Page 4 |
| **METHODS** | | |  |
| Eligibility criteria | 5 | Inclusion criteria are presented as follows: (1) study design is randomized controlled trial (RCT); (2) the frequency was > 1 Hz rTMS with the non-invasive stimulation and more than 10 sessions in clinical practice; (3) patients diagnosed with schizophrenia or schizoaffective disorder diagnosed according to standardized criteria such as Diagnostic and Statistical Manual of Mental Disorders (DSM), International Statistical Classification of Diseases and Related Health Problems (ICD) or MiniInternational Neuropsychiatric Interview. The severity of negative symptoms with schizophrenia were assessed with PANSS and the SANS (30). The severity of positive symptoms with schizophrenia PANSS, and depression with Schizophrenia were assessed with the Calgary Depression Scale (CDSS); (4) without comminating other psychiatric diseases. | Page 5 |
| Information sources | 6 | The systematic search was conducted on the electronic database including PubMed, EMBASE, Web of Science, SinoMed, PsycINFO and MEDLINE database for articles published from inception to July, 2023. | Page 4 |
| Search strategy | 7 | With the following keywords: “rTMS”, “repetitive transcranial magnetic stimulation”, “Schizophrenia”, “high-frequency” and “Negative Symptoms”. The reference lists of retrieved studies and relevant reviews were hand-searched, and the process mentioned above was repeatedly performed for ensuring that all eligible studies were included. | Page 5 |
| Selection process | 8 | The reference lists of retrieved studies and relevant reviews were hand-searched, and the process mentioned above was repeatedly performed for ensuring that all eligible studies were included. | Page 5 |
| Data collection process | 9 | Data were independently extracted by two investigators. Discrepancies were resolved by consensus. | Page 5 |
| Data items | 10a | (1) author, title, journal, publication year and study design; (2) the PANSS, the SANS and the CDSS rating scale values; (3) demographic and clinical characteristics were also extracted including. | Page 5 |
|  | 10b | Age, age of onset, duration of illness, gender distribution, antipsychotic medication, rTMS condition, frequency of treatment, follow up time and outcome measurements | Page 5 |
| Study risk of bias assessment | 11 | The Cochrane risk of bias was used by two investigators independently and with the differences resolved by discussion with all authors. The domain-based evaluation recommended by the Cochrane Handbook for Systematic Reviews of Intervention. | Page 6 |
| Effect measures | 12 | Cohen’s d standardized mean differences (SMD) were computed to calculate a random-effect meta-analysis with inverse variance weighting. In this meta-analysis, 95% confidence intervals (CIs) were calculated for categorical and continuous outcomes, significance level was set as P < 0.05. Publication bias of the primary outcome was evaluated using Funnel plots and Egger’s test. | Page 6 |
| Synthesis methods | 13a | The primary outcome was the efficacy of high-frequency rTMS group versus sham group on negative symptoms in patients diagnosed with schizophrenia. PANSS or SANS were consistently used as outcomes measurement. Severity of negative symptoms is positively correlated with level of score. The secondary outcomes were narrative analysis of the results from articles not included in the primary analysis, PANSS positive, and CDSS were used to evaluate the addition effective of high-frequency rTMS. | Page 5 |
|  | 13b | No missing summary statistics, or data conversions. | - |
|  | 13c | Cochrane RevMan software (version 5.3) and R software (version 4.3.1) were used to display results of individual studies and syntheses. | Page 6 |
|  | 13d | Cochrane RevMan software (version 5.3) and R software (version 4.3.1) were used to conduct the statistical analysis. Heterogeneity among studies was determined using the I2 test, with a P-value less than 0.1 and an I2 - value over 50%, suggesting the presence of significant study heterogeneity. | Page 6 |
|  | 13e | Four subgroup analyses were also conducted for further investigation | Page 6 |
|  | 13f | Sensitivity analysis was performed for evaluating the influence of a single study on the overall estimate by omitting one study. | Page 6 |
| Reporting bias assessment | 14 | Na | - |
| Certainty assessment | 15 | 95% confidence intervals (CIs) were calculated for categorical and continuous outcomes, significance level was set as P < 0.05. | Page 6 |
| **RESULTS** | | |  |
| Study selection | 16a | A systematic search was performed according to the PRISMA flow diagram. A total of 59 articles were identified after reviewing the literature, and 17 studies were ultimately included. | Page 7 |
|  | 16b | The reasons for exclusion were as follows: two studies focused on the efficacy of high-frequency rTMS for preventing cognition, two studies lacked endpoint results, four studies used drug combinations, two studies investigated the relationship between smoking and high-frequency rTMS, and several other studies focused on different topics | Page 7 |
| Study characteristics | 17 | Shown in the table 1. | - |
| Risk of bias in studies | 18 | Shown in the figure 1. | - |
| Results of individual studies | 19 |  | - |
| Results of syntheses | 20a | More than 15 sessions therapy span, frequency and location of the stimulation are contributing risk of bias. | - |
|  | 20b | Significant differences were found (SMD = -0.31; 95% CI = -0.49 – -0.14; P < 0.05) (Figure 2) between the high-frequency rTMS group and the sham group. A total of 791 participants were included in the analysis, and the endpoint was PANSS-negative scores. There was significant heterogeneity among the studies (I2 = 32%, P = 0.11). We also compared the high-frequency rTMS group versus the sham group in 10 studies using the SANS as the endpoint; the results revealed significant differences (SMD = -0.99; 95% CI = -1.64 to -0.33; P < 0.05) (Figure 3), with significant heterogeneity among the studies (I2 = 91%, P < 0.05). | Page 8 |
|  | 20c | We performed subgroup analysis to determine whether 15 sessions or more led to significant differences. The twelve included studies were carefully read. PANSS-negative scores were used to compare the efficacy of active versus sham rTMS. In total, 605 participants were included in this subgroup analysis. The significant effects were reported (SMD = -0.40; 95% CI = -0.57 – -0.24; P < 0.05), and there was no significant heterogeneity among the studies (I2 = 7%, P = 0.38) (Figure 5a).  Another subgroup analysis was conducted to investigate the effect of the frequency of rTMS. We found that 20 Hz (Figure 5b) (SMD = -0.48; 95% CI = -0.71 – -0.25; P<0.05) had a stronger effect than 10 Hz (Figure 5c) (SMD = -0.26; 95% CI = -0.49 – -0.02; P < 0.05). We found significant heterogeneity among the studies using 10 Hz (I2 = 33%, P = 0.17) and no significant heterogeneity among the studies using 20 Hz (I2 = 0%, P = 0.65).  An additional subgroup analysis was performed to determine the effect of the location of the stimulation. Thirteen studies were included in this subgroup analysis, and significant differences were observed (Figure 5d) (SMD = -0.34; 95% CI = -0.52 – -0.17; P < 0.05). There was low heterogeneity among the studies (I2 = 25%, P = 0.19). | Page 8 |
|  | 20d | No statistically significant differences were found (Figure 4) (SMD = -0.31; 95% CI = -0.49–0.14; P = 0.11). | Page 9 |
| Reporting biases | 21 | The funnel plot showed no asymmetry (Figure 6). Egger's regression test was performed to quantify the possible amount of bias, and the result was nonsignificant (P = 0.9605). Taken together, these complementary analyses indicate the absence of publication bias in this meta-analysis. | Page 9 |
| Certainty of evidence | 22 | No statistically significant differences were found (Figure 4) (SMD = -0.31; 95% CI = -0.49–0.14; P = 0.11). | Page 9 |
| **DISCUSSION** | | |  |
| Discussion | 23a | We conducted a meta-analysis of 17 randomized controlled trials that examined the use of high-frequency rTMS to treat negative symptoms in patients with schizophrenia. Among these studies, 15 included PANSS-negative scores, and 1 included only SANS scores. The SANS is a reliable scale for assessing negative symptoms in patients with schizophrenia. The 15 RCTs in our meta-analysis that included PANSS-negative scores suggested that high-frequency rTMS is beneficial for treating negative symptoms in schizophrenia patients. Additionally, 10 studies including SANS scores found a stronger effect than the studies using PANSS scores. Although the PANSS and SANS are widely used, their differences may result in variations in baseline data, different frequencies used in the experiments, and different stimulation locations. To further explore the effect of stimulation frequency on negative symptoms in schizophrenia patients, we conducted two subgroup analyses: one with a 10-Hz stimulation frequency (eight studies) and another with a 20-Hz stimulation frequency (six studies). Surprisingly, the studies using a 10-Hz frequency showed significant effects (P < 0.05), but the studies using a 20-Hz frequency showed stronger effects (P < 0.05). Numerous studies have recommended that more than three weeks of treatment may be necessary to achieve more significant effects, which means that 15 sessions or more could yield effective outcomes. Most studies recommend a treatment frequency of five times per week, and the duration of treatment increases with the number of treatments. In the 12 studies with treatment durations longer than three weeks, we observed only slight improvements, which demonstrated that an increase in duration did not result in stronger effects. | Page 10 |
|  | 23b | First, the heterogeneity of the baseline data, including age, sex, methods, specifications of the equipment, stimulation threshold and staging of illness, may have introduced bias in our results. Garg et al. found a more significant improvement in the sham intervention group by targeting the cerebellum, suggesting that the DLPFC may be an important target for schizophrenia-spectrum disorders | Page 11 |
|  | 23c | Second, the scoring system used herein is more subjective than the other scoring systems, which could introduce bias into the results. | Page 11 |
|  | 23d | We suggest that robust RCTs with large sample sizes and a standard protocol should be performed in the future to obtain more accurate data and verify our results. We recommend a frequency of 20 Hz, a number greater than 15 sessions, more significant benefits in the DLPFC. However, further large-scale randomized controlled trials, specific measurement scales for assessing negative symptoms in schizophrenia, standardized rTMS treatment protocols, and consistent outcome measures are needed to confirm the efficacy of rTMS in treating negative symptoms in schizophrenia patients. | Page 12 |
| **OTHER INFORMATION** | | |  |
| Registration and protocol | 24a | This systematic review and meta-analysis were registered in the International Prospective Register of Systematic Review (PROSPERO) trial registry (CRD42023450243). Ethical approval and patient consent were not required as all the analyses were based on previously published studies. | Page 4 |
|  | 24b | A protocol was not prepared. | - |
|  | 24c | Describe and explain any amendments to information provided at registration or in the protocol. | - |
| Support | 25 | Not available | - |
| Competing interests | 26 | The authors declare no competing interests. | - |
| Availability of data, code and other materials | 27 | Data extracted from included studies | - |

*From:*  Page MJ, McKenzie JE, Bossuyt PM, Boutron I, Hoffmann TC, Mulrow CD, et al. The PRISMA 2020 statement: an updated guideline for reporting systematic reviews. BMJ 2021;372:n71. doi: 10.1136/bmj.n71
