# Supplementary material for: The effects of high-frequency repetitive transcranial magnetic stimulation on negative symptoms in schizophrenia patients: A systemic review and meta-analysis
Source: PLoS One. 2025 Dec 11;20(12):e0337847. doi: 10.1371/journal.pone.0337847 (PMC12697975; doi:10.1371/journal.pone.0337847)
Supplement: S1 Table — (DOCX) [file pone.0337847.s002.docx]

| No. | Studies | Included | Reasons |
| --- | --- | --- | --- |
| 1 | Aydın, E. P., Genç, A., Dalkıran, M., Uyar, E. T., Deniz, İ., Özer, Ö. A., & Karamustafalıoğlu, K. O. (2018). Thioredoxin is not a marker for treatment-resistance depression but associated with cognitive function: An rTMS study. Progress in neuro-psychopharmacology & biological psychiatry, 80(Pt C), 322–328. https://doi.org/10.1016/j.pnpbp.2017.04.025 | N | Improper methodology |
| 2 | Barr, M. S., Farzan, F., Rusjan, P. M., Chen, R., Fitzgerald, P. B., & Daskalakis, Z. J. (2009). Potentiation of gamma oscillatory activity through repetitive transcranial magnetic stimulation of the dorsolateral prefrontal cortex. *Neuropsychopharmacology : official publication of the American College of Neuropsychopharmacology*, *34*(11), 2359–2367. https://doi.org/10.1038/npp.2009.79 | N | Cognition |
| 3 | Barr, M. S., Farzan, F., Tran, L. C., Fitzgerald, P. B., & Daskalakis, Z. J. (2012). A randomized controlled trial of sequentially bilateral prefrontal cortex repetitive transcranial magnetic stimulation in the treatment of negative symptoms in schizophrenia. Brain stimulation, 5(3), 337–346. https://doi.org/10.1016/j.brs.2011.06.003 | Y |  |
| 4 | Campana, M., Schneider-Axmann, T., Wobrock, T., Malchow, B., Langguth, B., Landgrebe, M., Eichhammer, P., Frank, E., Cordes, J., Wölwer, W., Gaebel, W., Winterer, G., Hajak, G., Ohmann, C., Verde, P. E., Rietschel, M., Ahmed, R., Mortazavi, M., Strube, W., Falkai, P., … Wagner, E. (2024). Assessing the impact of sex on high-frequency repetitive transcranial magnetic stimulation´s clinical response in schizophrenia - results from a secondary analysis. *The world journal of biological psychiatry : the official journal of the World Federation of Societies of Biological Psychiatry*, *25*(4), 233–241. https://doi.org/10.1080/15622975.2024.2327028 | N | irrelevant |
| 5 | de Weijer, A. D., Sommer, I. E., Lotte Meijering, A., Bloemendaal, M., Neggers, S. F., Daalman, K., & Boezeman, E. H. (2014). High frequency rTMS; a more effective treatment for auditory verbal hallucinations?. *Psychiatry research*, *224*(3), 204–210. https://doi.org/10.1016/j.pscychresns.2014.10.007 | N | Auditory verbal hallucinations |
| 6 | Dlabac-de Lange, J. J., Liemburg, E. J., Bais, L., van de Poel-Mustafayeva, A. T., de Lange-de Klerk, E. S. M., Knegtering, H., & Aleman, A. (2017). Effect of Bilateral Prefrontal rTMS on Left Prefrontal NAA and Glx Levels in Schizophrenia Patients with Predominant Negative Symptoms: An Exploratory Study. *Brain stimulation*, *10*(1), 59–64. https://doi.org/10.1016/j.brs.2016.08.002 | N | Low-frequency |
| 7 | Dollfus, S., Jaafari, N., Guillin, O., Trojak, B., Plaze, M., Saba, G., Nauczyciel, C., Montagne Larmurier, A., Chastan, N., Meille, V., Krebs, M. O., Ayache, S. S., Lefaucheur, J. P., Razafimandimby, A., Leroux, E., Morello, R., Marie Batail, J., Brazo, P., Lafay, N., Wassouf, I., … Etard, O. (2018). High-Frequency Neuronavigated rTMS in Auditory Verbal Hallucinations: A Pilot Double-Blind Controlled Study in Patients With Schizophrenia. *Schizophrenia bulletin*, *44*(3), 505–514. https://doi.org/10.1093/schbul/sbx127 | N | Auditory verbal hallucinations |
| 8 | Fitzgerald, P. B., Herring, S., Hoy, K., McQueen, S., Segrave, R., Kulkarni, J., & Daskalakis, Z. J. (2008). A study of the effectiveness of bilateral transcranial magnetic stimulation in the treatment of the negative symptoms of schizophrenia. *Brain stimulation*, *1*(1), 27–32. https://doi.org/10.1016/j.brs.2007.08.001 | Y |  |
| 9 | Garg, S., Sinha, V. K., Tikka, S. K., Mishra, P., & Goyal, N. (2016). The efficacy of cerebellar vermal deep high frequency (theta range) repetitive transcranial magnetic stimulation (rTMS) in schizophrenia: A randomized rater blind-sham controlled study. *Psychiatry research*, *243*, 413–420. https://doi.org/10.1016/j.psychres.2016.07.023 | Y |  |
| 10 | Guse, B., Falkai, P., Gruber, O., Whalley, H., Gibson, L., Hasan, A., Obst, K., Dechent, P., McIntosh, A., Suchan, B., & Wobrock, T. (2013). The effect of long-term high frequency repetitive transcranial magnetic stimulation on working memory in schizophrenia and healthy controls--a randomized placebo-controlled, double-blind fMRI study. *Behavioural brain research*, *237*, 300–307. https://doi.org/10.1016/j.bbr.2012.09.034 | N | Working memory |
| 11 | Hansbauer, M., Wobrock, T., Kunze, B., Langguth, B., Landgrebe, M., Eichhammer, P., Frank, E., Cordes, J., Wölwer, W., Winterer, G., Gaebel, W., Hajak, G., Ohmann, C., Verde, P. E., Rietschel, M., Ahmed, R., Honer, W. G., Malchow, B., Strube, W., Schneider-Axmann, T., … Hasan, A. (2018). Efficacy of high-frequency repetitive transcranial magnetic stimulation on PANSS factors in schizophrenia with predominant negative symptoms - Results from an exploratory re-analysis. *Psychiatry research*, *263*, 22–29. https://doi.org/10.1016/j.psychres.2018.02.030 | N | irrelevant |
| 12 | Jaiswal, N., Kar, S. K., & Gupta, P. K. (2022). Comments on "High frequency repetitive transcranial magnetic stimulation of dorsomedial prefrontal cortex for negative symptoms in patients with schizophrenia: A double-blind, randomized controlled trial". *Psychiatry research*, *312*, 114532. https://doi.org/10.1016/j.psychres.2022.114532 | N | Comments |
| 13 | Kamp, D., Brinkmeyer, J., Agelink, M. W., Habakuck, M., Mobascher, A., Wölwer, W., & Cordes, J. (2016). High frequency repetitive transcranial magnetic stimulation (rTMS) reduces EEG-hypofrontality in patients with schizophrenia. *Psychiatry research*, *236*, 199–201. https://doi.org/10.1016/j.psychres.2016.01.007 | N | EEG-hypofrontality |
| 14 | Kamp, D., Engelke, C., Wobrock, T., Wölwer, W., Winterer, G., Schmidt-Kraepelin, C., Gaebel, W., Langguth, B., Landgrebe, M., Eichhammer, P., Frank, E., Hajak, G., Ohmann, C., Verde, P. E., Rietschel, M., Raees, A., Honer, W. G., Malchow, B., Schneider-Axmann, T., Falkai, P., … Cordes, J. (2019). Left prefrontal high-frequency rTMS may improve movement disorder in schizophrenia patients with predominant negative symptoms - A secondary analysis of a sham-controlled, randomized multicenter trial. *Schizophrenia research*, *204*, 445–447. https://doi.org/10.1016/j.schres.2018.09.017 | N | Movement disorder |
| 15 | Kimura, H., Kanahara, N., Takase, M., Yoshida, T., Watanabe, H., & Iyo, M. (2016). A randomized, sham-controlled study of high frequency rTMS for auditory hallucination in schizophrenia. *Psychiatry research*, *241*, 190–194. https://doi.org/10.1016/j.psychres.2016.04.119 | N | Auditory verbal hallucinations |
| 16 | Kozak, K., Sharif-Razi, M., Morozova, M., Gaudette, E. V., Barr, M. S., Daskalakis, Z. J., Blumberger, D. M., & George, T. P. (2018). Effects of short-term, high-frequency repetitive transcranial magnetic stimulation to bilateral dorsolateral prefrontal cortex on smoking behavior and cognition in patients with schizophrenia and non-psychiatric controls. *Schizophrenia research*, *197*, 441–443. https://doi.org/10.1016/j.schres.2018.02.015 | N | Smoking behavior and cognition |
| 17 | Kumar, N., Vishnubhatla, S., Wadhawan, A. N., Minhas, S., & Gupta, P. (2020). A randomized, double blind, sham-controlled trial of repetitive transcranial magnetic stimulation (rTMS) in the treatment of negative symptoms in schizophrenia. Brain stimulation, 13(3), 840–849. https://doi.org/10.1016/j.brs.2020.02.016 | Y |  |
| 18 | Mittrach, M., Thünker, J., Winterer, G., Agelink, M. W., Regenbrecht, G., Arends, M., Mobascher, A., Kim, S. J., Wölwer, W., Brinkmeyer, J., Gaebel, W., & Cordes, J. (2010). The tolerability of rTMS treatment in schizophrenia with respect to cognitive function. *Pharmacopsychiatry*, *43*(3), 110–117. https://doi.org/10.1055/s-0029-1242824 | N | Cognitive function |
| 19 | Montagne-Larmurier, A., Etard, O., Razafimandimby, A., Morello, R., & Dollfus, S. (2009). Two-day treatment of auditory hallucinations by high frequency rTMS guided by cerebral imaging: a 6 month follow-up pilot study. *Schizophrenia research*, *113*(1), 77–83. https://doi.org/10.1016/j.schres.2009.05.006 | N | Auditory verbal hallucinations |
| 20 | Nathou, C., Etard, O., Simon, G., & Dollfus, S. (2015). How do high- and low-frequency repetitive transcranial magnetic stimulations modulate the temporal cortex. *Psychophysiology*, *52*(2), 192–198. https://doi.org/10.1111/psyp.12323 | N | Irrelevant |
| 21 | Prikryl, R., Ustohal, L., Kucerova, H. P., Kasparek, T., Jarkovsky, J., Hublova, V., Vrzalova, M., & Ceskova, E. (2014). Repetitive transcranial magnetic stimulation reduces cigarette consumption in schizophrenia patients. *Progress in neuro-psychopharmacology & biological psychiatry*, *49*, 30–35. https://doi.org/10.1016/j.pnpbp.2013.10.019 | N | Smoking behavior |
| 22 | Prikryl, R., Mikl, M., Prikrylova Kucerová, H., Ustohal, L., Kasparek, T., Marecek, R., Vrzalova, M., Ceskova, E., & Vanicek, J. (2012). Does repetitive transcranial magnetic stimulation have a positive effect on working memory and neuronal activation in treatment of negative symptoms of schizophrenia?. *Neuro endocrinology letters*, *33*(1), 90–97. | N | working memory |
| 23 | Prikryl, R., Ustohal, L., Prikrylova Kucerova, H., Kasparek, T., Venclikova, S., Vrzalova, M., & Ceskova, E. (2013). A detailed analysis of the effect of repetitive transcranial magnetic stimulation on negative symptoms of schizophrenia: a double-blind trial. *Schizophrenia research*, *149*(1-3), 167–173. https://doi.org/10.1016/j.schres.2013.06.015 | Y |  |
| 24 | Quan, W. X., Zhu, X. L., Qiao, H., Zhang, W. F., Tan, S. P., Zhou, D. F., & Wang, X. Q. (2015). The effects of high-frequency repetitive transcranial magnetic stimulation (rTMS) on negative symptoms of schizophrenia and the follow-up study. Neuroscience letters, 584, 197–201. https://doi.org/10.1016/j.neulet.2014.10.029 | Y |  |
| 25 | Wing, V. C., Bacher, I., Wu, B. S., Daskalakis, Z. J., & George, T. P. (2012). High frequency repetitive transcranial magnetic stimulation reduces tobacco craving in schizophrenia. *Schizophrenia research*, *139*(1-3), 264–266. https://doi.org/10.1016/j.schres.2012.03.006 | N | tobacco craving |
| 26 | Wobrock, T., Guse, B., Cordes, J., Wölwer, W., Winterer, G., Gaebel, W., Langguth, B., Landgrebe, M., Eichhammer, P., Frank, E., Hajak, G., Ohmann, C., Verde, P. E., Rietschel, M., Ahmed, R., Honer, W. G., Malchow, B., Schneider-Axmann, T., Falkai, P., & Hasan, A. (2015). Left prefrontal high-frequency repetitive transcranial magnetic stimulation for the treatment of schizophrenia with predominant negative symptoms: a sham-controlled, randomized multicenter trial. *Biological psychiatry*, *77*(11), 979–988. https://doi.org/10.1016/j.biopsych.2014.10.009 | Y |  |
| 27 | Wölwer, W., Lowe, A., Brinkmeyer, J., Streit, M., Habakuck, M., Agelink, M. W., Mobascher, A., Gaebel, W., & Cordes, J. (2014). Repetitive transcranial magnetic stimulation (rTMS) improves facial affect recognition in schizophrenia. *Brain stimulation*, *7*(4), 559–563. https://doi.org/10.1016/j.brs.2014.04.011 | N | recognition |
| 28 | Yang, L. L., Zhao, D., Kong, L. L., Sun, Y. Q., Wang, Z. Y., Gao, Y. Y., Li, N., Lu, L., Shi, L., Wang, X. Y., & Wang, Y. M. (2019). High-frequency repetitive transcranial magnetic stimulation (rTMS) improves neurocognitive function in bipolar disorder. *Journal of affective disorders*, *246*, 851–856. https://doi.org/10.1016/j.jad.2018.12.102 | N | bipolar disorder |
| 29 | Chang, C. C., Lin, Y. Y., Tzeng, N. S., Kao, Y. C., & Chang, H. A. (2021). Adjunct high-frequency transcranial random noise stimulation over the lateral prefrontal cortex improves negative symptoms of schizophrenia: A randomized, double-blind, sham-controlled pilot study. *Journal of psychiatric research*, *132*, 151–160. https://doi.org/10.1016/j.jpsychires.2020.10.008 | N | Noise stimulation |
| 30 | Fuggetta, G., & Noh, N. A. (2013). A neurophysiological insight into the potential link between transcranial magnetic stimulation, thalamocortical dysrhythmia and neuropsychiatric disorders. *Experimental neurology*, *245*, 87–95. https://doi.org/10.1016/j.expneurol.2012.10.010 | N | Irrelevant |
| 31 | Gan, H., Zhu, J., Zhuo, K., Zhang, J., Tang, Y., Qian, Z., Xiang, Q., Li, X., Zhu, Y., Wang, J., Wang, J., & Liu, D. (2021). High frequency repetitive transcranial magnetic stimulation of dorsomedial prefrontal cortex for negative symptoms in patients with schizophrenia: A double-blind, randomized controlled trial. *Psychiatry research*, *299*, 113876. https://doi.org/10.1016/j.psychres.2021.113876 | N | Lack statistics |
| 32 | Goyal, N., Nizamie, S. H., & Desarkar, P. (2007). Efficacy of adjuvant high frequency repetitive transcranial magnetic stimulation on negative and positive symptoms of schizophrenia: preliminary results of a double-blind sham-controlled study. *The Journal of neuropsychiatry and clinical neurosciences*, *19*(4), 464–467. https://doi.org/10.1176/jnp.2007.19.4.464 | N | Improper methodology |
| 33 | Hajak, G., Marienhagen, J., Langguth, B., Werner, S., Binder, H., & Eichhammer, P. (2004). High-frequency repetitive transcranial magnetic stimulation in schizophrenia: a combined treatment and neuroimaging study. *Psychological medicine*, *34*(7), 1157–1163. https://doi.org/10.1017/s0033291704002338 | N | Lack statistics |
| 34 | Holi, M. M., Eronen, M., Toivonen, K., Toivonen, P., Marttunen, M., & Naukkarinen, H. (2004). Left prefrontal repetitive transcranial magnetic stimulation in schizophrenia. Schizophrenia bulletin, 30(2), 429–434. https://doi.org/10.1093/oxfordjournals.schbul.a007089 | Y |  |
| 35 | Huber, T. J., Schneider, U., & Rollnik, J. (2003). Gender differences in the effect of repetitive transcranial magnetic stimulation in schizophrenia. *Psychiatry research*, *120*(1), 103–105. https://doi.org/10.1016/s0165-1781(03)00170-7 | N | Irrelevant |
| 36 | Novák, T., Horácek, J., Mohr, P., Kopecek, M., Skrdlantová, L., Klirova, M., Rodriguez, M., Spaniel, F., Dockery, C., & Höschl, C. (2006). The double-blind sham-controlled study of high-frequency rTMS (20 Hz) for negative symptoms in schizophrenia: negative results. *Neuro endocrinology letters*, *27*(1-2), 209–213. | N | Lack statistics |
| 37 | Prikryl, R., Kasparek, T., Skotakova, S., Ustohal, L., Kucerova, H., & Ceskova, E. (2007). Treatment of negative symptoms of schizophrenia using repetitive transcranial magnetic stimulation in a double-blind, randomized controlled study. *Schizophrenia research*, *95*(1-3), 151–157. https://doi.org/10.1016/j.schres.2007.06.019 | N | Lack statistics |
| 38 | Rollnik, J. D., Huber, T. J., Mogk, H., Siggelkow, S., Kropp, S., Dengler, R., Emrich, H. M., & Schneider, U. (2000). High frequency repetitive transcranial magnetic stimulation (rTMS) of the dorsolateral prefrontal cortex in schizophrenic patients. *Neuroreport*, *11*(18), 4013–4015. https://doi.org/10.1097/00001756-200012180-00022 | N | Lack statistics |
| 39 | Sachdev, P., Loo, C., Mitchell, P., & Malhi, G. (2005). Transcranial magnetic stimulation for the deficit syndrome of schizophrenia: a pilot investigation. *Psychiatry and clinical neurosciences*, *59*(3), 354–357. https://doi.org/10.1111/j.1440-1819.2005.01382.x | N | deficit syndrome |
| 40 | Schneider, A. L., Schneider, T. L., & Stark, H. (2008). Repetitive transcranial magnetic stimulation (rTMS) as an augmentation treatment for the negative symptoms of schizophrenia: a 4-week randomized placebo controlled study. *Brain stimulation*, *1*(2), 106–111. https://doi.org/10.1016/j.brs.2008.01.001 | N | Improper methodology |
| 41 | Wagner, E., Wobrock, T., Kunze, B., Langguth, B., Landgrebe, M., Eichhammer, P., Frank, E., Cordes, J., Wölwer, W., Winterer, G., Gaebel, W., Hajak, G., Ohmann, C., Verde, P. E., Rietschel, M., Ahmed, R., Honer, W. G., Siskind, D., Malchow, B., Strube, W., … Hasan, A. (2019). Efficacy of high-frequency repetitive transcranial magnetic stimulation in schizophrenia patients with treatment-resistant negative symptoms treated with clozapine. *Schizophrenia research*, *208*, 370–376. https://doi.org/10.1016/j.schres.2019.01.021 | N | Lack ending statistics |
| 42 | Xue, F., Wang, X. F., Kong, F. N., Yin, T. L., Wang, Y. H., Shi, L. D., Liu, X. W., Yu, H. J., Liu, L. J., Zhu, P., Qi, X. X., Xu, X. J., Hu, H. P., & Li, S. X. (2024). Effects of bilateral repetitive transcranial magnetic stimulation on prospective memory in patients with schizophrenia: A double-blind randomized controlled clinical trial. *Neuropsychopharmacology reports*, *44*(1), 97–108. https://doi.org/10.1002/npr2.12397 | N | Lack ending statistics |
| 43 | A comparative study of high frequency repetitive transcranial magnetic stimulation to treat negative symptoms in patients with schizophrenia．LIU Lihua，XUE Xiaobin，LU Xiaozi，et a1．Qingdao Mental Health Cen ter，Qingdao 266034， China | Y |  |
| 44 | Li, Shao-Mei, Liang, Chang-Kuen & Tang, Jing-Hsiung. (2018). Effectiveness and safety of high-frequency repetitive transcranial magnetic stimulation (rTMS) for the treatment of negative symptoms of schizophrenia. Baiqiu'en Medical Journal,16(06),580-581. doi:10.16485/j.issn.2095-7858.2018.06.020. (in Chinese) | Y |  |
| 45 | LI Zhaohui, WANG Jiang, CAI Guangchao. Efficacy and safety analysis of high-frequency repetitive transcranial magnetic stimulation for the treatment of chronic schizophrenia with negative symptoms[J]. Hebei Medicine,2017,39(15):2326-2328. (in Chinese) | Y |  |
| 46 | HUANG Shaoya, CHEN Xuxian, LIN Lixin, et al. Effects of long-range high-frequency repetitive transcranial magnetic stimulation on negative symptoms of schizophrenia[J]. Journal of Qiqihar Medical College,2019,40(17):2128-2130. (in Chinese) | Y |  |
| 47 | Li-Shin Lin, Shao-Ya Huang, Feng Lin, et al. Observation on the efficacy of high-frequency repetitive transcranial magnetic stimulation for the treatment of negative symptoms of schizophrenia[J]. Journal of Clinical Rational Medication,2018,11(19):129-130.DOI:10.15887/j.cnki.13-1389/r.2018.19.072. (in Chinese) | Y |  |
| 48 | Du, X. D., Li, Z., Yuan, N., Yin, M., Zhao, X. L., Lv, X. L., Zou, S. Y., Zhang, J., Zhang, G. Y., Li, C. W., Pan, H., Yang, L., Wu, S. Q., Yue, Y., Wu, Y. X., & Zhang, X. Y. (2022). Delayed improvements in visual memory task performance among chronic schizophrenia patients after high-frequency repetitive transcranial magnetic stimulation. *World journal of psychiatry*, *12*(9), 1169–1182. https://doi.org/10.5498/wjp.v12.i9.1169 | Y |  |
| 49 | Singh, S., Kumar, N., Verma, R., & Nehra, A. (2020). The safety and efficacy of adjunctive 20-Hz repetitive transcranial magnetic stimulation for treatment of negative symptoms in patients with schizophrenia: A double-blinded, randomized, sham-controlled study. *Indian journal of psychiatry*, *62*(1), 21–29. https://doi.org/10.4103/psychiatry.IndianJPsychiatry_361_19 | Y |  |
| 50 | Wen, N., Chen, L., Miao, X., Zhang, M., Zhang, Y., Liu, J., Xu, Y., Tong, S., Tang, W., Wang, M., Liu, J., Zhou, S., Fang, X., & Zhao, K. (2021). Effects of High-Frequency rTMS on Negative Symptoms and Cognitive Function in Hospitalized Patients With Chronic Schizophrenia: A Double-Blind, Sham-Controlled Pilot Trial. *Frontiers in psychiatry*, *12*, 736094. https://doi.org/10.3389/fpsyt.2021.736094 | Y |  |
| 51 | Xiu, M. H., Guan, H. Y., Zhao, J. M., Wang, K. Q., Pan, Y. F., Su, X. R., Wang, Y. H., Guo, J. M., Jiang, L., Liu, H. Y., Sun, S. G., Wu, H. R., Geng, H. S., Liu, X. W., Yu, H. J., Wei, B. C., Li, X. P., Trinh, T., Tan, S. P., & Zhang, X. Y. (2020). Cognitive Enhancing Effect of High-Frequency Neuronavigated rTMS in Chronic Schizophrenia Patients With Predominant Negative Symptoms: A Double-Blind Controlled 32-Week Follow-up Study. *Schizophrenia bulletin*, *46*(5), 1219–1230. https://doi.org/10.1093/schbul/sbaa035 | Y |  |
| 52 | Hasan, A., Wobrock, T., Guse, B., Langguth, B., Landgrebe, M., Eichhammer, P., Frank, E., Cordes, J., Wölwer, W., Musso, F., Winterer, G., Gaebel, W., Hajak, G., Ohmann, C., Verde, P. E., Rietschel, M., Ahmed, R., Honer, W. G., Dechent, P., Malchow, B., … Koutsouleris, N. (2017). Structural brain changes are associated with response of negative symptoms to prefrontal repetitive transcranial magnetic stimulation in patients with schizophrenia. Molecular psychiatry, 22(6), 857–864. https://doi.org/10.1038/mp.2016.161 | N | Irrelevant |
| 53 | Mehta, U. M., Naik, S. S., Thanki, M. V., & Thirthalli, J. (2019). Investigational and Therapeutic Applications of Transcranial Magnetic Stimulation in Schizophrenia. Current psychiatry reports, 21(9), 89. https://doi.org/10.1007/s11920-019-1076-2 | N | Irrelevant |
| 54 | Lai, I. C., Yang, C. C., Kuo, T. B., & Shieh, K. R. (2010). Transcranial magnetic stimulation for auditory hallucination in severe schizophrenia: partial efficacy and acute elevation of sympathetic modulation. *Psychiatry and clinical neurosciences*, *64*(3), 333–335. https://doi.org/10.1111/j.1440-1819.2010.02078.x | N | Auditory hallucination |
| 55 | Yang, L. L., Zhao, D., Kong, L. L., Sun, Y. Q., Wang, Z. Y., Gao, Y. Y., Li, N., Lu, L., Shi, L., Wang, X. Y., & Wang, Y. M. (2019). High-frequency repetitive transcranial magnetic stimulation (rTMS) improves neurocognitive function in bipolar disorder. *Journal of affective disorders*, *246*, 851–856. https://doi.org/10.1016/j.jad.2018.12.102 | N | Neurocognitive function |
| 56 | Bilek, E., Schäfer, A., Ochs, E., Esslinger, C., Zangl, M., Plichta, M. M., Braun, U., Kirsch, P., Schulze, T. G., Rietschel, M., Meyer-Lindenberg, A., & Tost, H. (2013). Application of high-frequency repetitive transcranial magnetic stimulation to the DLPFC alters human prefrontal-hippocampal functional interaction. *The Journal of neuroscience : the official journal of the Society for Neuroscience*, *33*(16), 7050–7056. https://doi.org/10.1523/JNEUROSCI.3081-12.2013 | N | Irrelevant |
| 57 | Campana, M., Wagner, E., Wobrock, T., Langguth, B., Landgrebe, M., Eichhammer, P., Frank, E., Cordes, J., Wölwer, W., Winterer, G., Gaebel, W., Hajak, G., Ohmann, C., Verde, P. E., Rietschel, M., Malchow, B., Ahmed, R., Strube, W., Häckert, J., Schneider-Axmann, T., … Hasan, A. (2021). Effects of high-frequency prefrontal rTMS on heart frequency rates and blood pressure in schizophrenia. *Journal of psychiatric research*, *140*, 243–249. https://doi.org/10.1016/j.jpsychires.2021.06.010 | N | Irrelevant |
| 58 | Ray, P., Sinha, V. K., & Tikka, S. K. (2015). Adjuvant low-frequency rTMS in treating auditory hallucinations in recent-onset schizophrenia: a randomized controlled study investigating the effect of high-frequency priming stimulation. *Annals of general psychiatry*, *14*, 8. https://doi.org/10.1186/s12991-015-0046-2 | N | Auditory hallucinations |
| 59 | Garg, S., Tikka, S. K., Goyal, N., Sinha, V. K., & Nizamie, S. H. (2013). Amelioration of anergia and thought disorder with adjunctive high frequency cerebellar vermal repetitive transcranial magnetic stimulation in schizophrenia: a case report. *Schizophrenia research*, *143*(1), 225–227. https://doi.org/10.1016/j.schres.2012.10.022 | N | case report |
