# Supplementary material for: The effects of high-frequency repetitive transcranial magnetic stimulation on negative symptoms in schizophrenia patients: A systemic review and meta-analysis
Source: PLoS One. 2025 Dec 11;20(12):e0337847. doi: 10.1371/journal.pone.0337847 (PMC12697975; doi:10.1371/journal.pone.0337847)
Supplement: S2 Table — (DOCX) [file pone.0337847.s003.docx]

**All data is extracted by Boxing Wang and Xinyue Zhu.**

| **m1=mean active** |
| --- |
| **s1=SD** |
| **n1=active total** |
| **m2=mean control** |
| **s1=SD** |
| **n1=control total** |

**PANSS Negative symptom**

| Studies | year | m1 | s1 | n1 | m2 | s2 | n2 |
| --- | --- | --- | --- | --- | --- | --- | --- |
| Barr et al. | 2012 | 14 | 6.08 | 13 | 14.17 | 4.84 | 12 |
| Du et al. | 2022 | 22.8 | 8.8 | 20 | 22.6 | 7.5 | 18 |
| Fitzgerald et al. | 2008 | 15.5 | 3.1 | 10 | 17.9 | 5.5 | 10 |
| Garg et al. | 2016 | 20.35 | 7.93 | 20 | 17.55 | 7.85 | 20 |
| Holi et al. | 2004 | 27.5 | 10.9 | 11 | 25.2 | 5.8 | 11 |
| Kumar et al. | 2020 | 18.5 | 5.4 | 50 | 20.8 | 4.89 | 50 |
| Liu et al. | 2017 | 18.47 | 2.55 | 20 | 20.95 | 3.14 | 20 |
| Li et al. | 2017 | 20.36 | 4.31 | 25 | 23.88 | 3.56 | 25 |
| Li et al. | 2018 | 18.74 | 2.1 | 20 | 20.85 | 4.31 | 21 |
| Quan et al. | 2015 | 20.03 | 5.04 | 78 | 22.59 | 5.79 | 39 |
| Singh et al. | 2020 | 18.93 | 4.63 | 15 | 22.4 | 5.12 | 15 |
| Wen et al. | 2021 | 24.5 | 3.9 | 23 | 25.6 | 3.1 | 22 |
| Wobrock et al. | 2015 | 22.7 | 6.1 | 62 | 22.7 | 5.8 | 64 |
| Xiu et al. 10Hz | 2020 | 21.8 | 5.8 | 32 | 22.9 | 10.5 | 30 |
| Xiu et al. 20Hz | 2020 | 20.1 | 5.1 | 35 | 22.9 | 10.5 | 30 |

**More than 15 sessions**

| **Studies** | **year** | **m1** | **s1** | **n1** | **m2** | **s2** | **n2** |
| --- | --- | --- | --- | --- | --- | --- | --- |
| Barr et al. | 2012 | 14 | 6.08 | 13 | 14.17 | 4.84 | 12 |
| Du et al. | 2022 | 22.8 | 8.8 | 20 | 22.6 | 7.5 | 18 |
| Holi et al. | 2004 | 27.5 | 10.9 | 11 | 25.2 | 5.8 | 11 |
| Kumar et al. | 2020 | 18.5 | 5.4 | 50 | 20.8 | 4.89 | 50 |
| Li et al. | 2017 | 20.36 | 4.31 | 25 | 23.88 | 3.56 | 25 |
| Li et al. | 2018 | 18.74 | 2.1 | 20 | 20.85 | 4.31 | 21 |
| Liu et al. | 2017 | 18.47 | 2.55 | 20 | 20.95 | 3.14 | 20 |
| Quan et al. | 2015 | 20.03 | 5.04 | 78 | 22.59 | 5.79 | 39 |
| Singh et al. | 2020 | 18.93 | 4.63 | 15 | 22.4 | 5.12 | 15 |
| Wen et al. | 2021 | 24.5 | 3.9 | 23 | 25.6 | 3.1 | 22 |
| Xiu et al. 10Hz | 2020 | 21.8 | 5.8 | 32 | 22.9 | 10.5 | 30 |
| Xiu et al. 20Hz | 2020 | 20.1 | 5.1 | 35 | 22.9 | 10.5 | 30 |

**20 Hz group**

| **Studies** | **year** | **m1** | **s1** | **n1** | **m2** | **s2** | **n2** |
| --- | --- | --- | --- | --- | --- | --- | --- |
| Barr et al. | 2012 | 14 | 6.08 | 13 | 14.17 | 4.84 | 12 |
| Kumar et al. | 2020 | 18.5 | 5.4 | 50 | 20.8 | 4.89 | 50 |
| Li et al. | 2018 | 18.74 | 2.1 | 20 | 20.85 | 4.31 | 21 |
| Liu et al. | 2017 | 18.47 | 2.55 | 20 | 20.95 | 3.14 | 20 |
| Singh et al. | 2020 | 18.93 | 4.63 | 15 | 22.4 | 5.12 | 15 |
| Xiu et al. 20Hz | 2020 | 20.1 | 5.1 | 35 | 22.9 | 10.5 | 30 |

**10 Hz group**

| **Studies** | **year** | **m1** | **s1** | **n1** | **m2** | **s2** | **n2** |
| --- | --- | --- | --- | --- | --- | --- | --- |
| Du et al. | 2022 | 22.8 | 8.8 | 20 | 22.6 | 7.5 | 18 |
| Fitzgerald et al. | 2008 | 15.5 | 3.1 | 10 | 17.9 | 5.5 | 10 |
| Holi et al. | 2004 | 27.5 | 10.9 | 11 | 25.2 | 5.8 | 11 |
| Li et al. 2017 | 2017 | 20.36 | 4.31 | 25 | 23.88 | 3.56 | 25 |
| Quan et al. | 2015 | 20.03 | 5.04 | 78 | 22.59 | 5.79 | 39 |
| Wen et al. | 2021 | 24.5 | 3.9 | 23 | 25.6 | 3.1 | 22 |
| Wobrock et al. |  | 22.7 | 6.1 | 62 | 22.7 | 5.8 | 64 |
| Xiu et al. 10Hz | 2020 | 21.8 | 5.8 | 32 | 22.9 | 10.5 | 30 |

**DLPFC group**

| **Studies** | **year** | **m1** | **s1** | **n1** | **m2** | **s2** | **n2** |
| --- | --- | --- | --- | --- | --- | --- | --- |
| Barr et al. | 2012 | 14 | 6.08 | 13 | 14.17 | 4.84 | 12 |
| Du et al. | 2022 | 22.8 | 8.8 | 20 | 22.6 | 7.5 | 18 |
| Holi et al. | 2004 | 27.5 | 10.9 | 11 | 25.2 | 5.8 | 11 |
| Kumar et al. | 2020 | 18.5 | 5.4 | 50 | 20.8 | 4.89 | 50 |
| Li et al. 2017 | 2017 | 20.36 | 4.31 | 25 | 23.88 | 3.56 | 25 |
| Li et al. 2018 | 2018 | 18.74 | 2.1 | 20 | 20.85 | 4.31 | 21 |
| Liu et al. | 2017 | 18.47 | 2.55 | 20 | 20.95 | 3.14 | 20 |
| Quan et al. | 2015 | 20.03 | 5.04 | 78 | 22.59 | 5.79 | 39 |
| Singh et al. | 2020 | 18.93 | 4.63 | 15 | 22.4 | 5.12 | 15 |
| Wen et al. | 2021 | 24.5 | 3.9 | 23 | 25.6 | 3.1 | 22 |
| Wobrock et al. | 2015 | 22.7 | 6.1 | 62 | 22.7 | 5.8 | 64 |
| Xiu et al. 10Hz | 2020 | 21.8 | 5.8 | 32 | 22.9 | 10.5 | 30 |
| Xiu et al. 20Hz | 2020 | 20.1 | 5.1 | 35 | 22.9 | 10.5 | 30 |

**PANSS Positive symptom**

| **Studies** | **year** | **m1** | **s1** | **n1** | **m2** | **s2** | **n2** |
| --- | --- | --- | --- | --- | --- | --- | --- |
| Barr et al. | 2012 | 13 | 4.26 | 13 | 13.22 | 4.21 | 12 |
| Du et al. | 2022 | 12.5 | 4 | 20 | 10.3 | 3.6 | 18 |
| Garg et al. | 2016 | 24.35 | 6.3 | 20 | 22.45 | 5.78 | 20 |
| Holi et al. | 2004 | 20 | 9.1 | 11 | 19.1 | 7.4 | 11 |
| Kumar et al. | 2020 | 8.5 | 1.85 | 50 | 8.7 | 2.12 | 50 |
| Li et al. 2017 | 2017 | 13.56 | 3.9 | 25 | 12.8 | 4.5 | 25 |
| Li et al. 2018 | 2018 | 14.56 | 4.3 | 20 | 17.17 | 4.1 | 21 |
| Liu et al. | 2017 | 13.95 | 2.44 | 20 | 15.8 | 3.07 | 20 |
| Quan et al. | 2015 | 10.85 | 4.78 | 78 | 11.13 | 4.72 | 39 |
| Singh et al. | 2020 | 8.86 | 2.77 | 15 | 8.2 | 1.69 | 15 |
| Wen et al. | 2021 | 16.7 | 2.8 | 23 | 16.9 | 4.2 | 22 |
| Wobrock et al. | 2015 | 12.4 | 4.1 | 62 | 12.4 | 4.6 | 64 |
| Xiu et al. 10Hz | 2020 | 11 | 5.7 | 32 | 10.4 | 2.2 | 30 |
| Xiu et al. 20Hz | 2020 | 10.1 | 4.1 | 35 | 10.4 | 2.2 | 30 |

**CDSS**

| **Studies** | **year** | **m1** | **s1** | **n1** | **m2** | **s2** | **n2** |
| --- | --- | --- | --- | --- | --- | --- | --- |
| Barr et al. | 2012 | 2.38 | 2.06 | 13 | 1.67 | 1.92 | 12 |
| Garg et al. | 2016 | 5.2 | 3.25 | 20 | 5.7 | 3.57 | 20 |
| Kumar et al. | 2020 | 0.12 | 0.44 | 50 | 0.12 | 0.72 | 50 |
| Prikryl et al. | 2013 | 0.04 | 0.21 | 23 | 0.76 | 1.48 | 17 |
| Singh et al. | 2020 | 1.33 | 0.97 | 15 | 1.33 | 1.23 | 15 |
| Wobrock et al. | 2015 | 4.4 | 3.5 | 62 | 4.6 | 4.4 | 64 |

**SANS**

| **Studies** | **year** | **m1** | **s1** | **n1** | **m2** | **s2** | **n2** |
| --- | --- | --- | --- | --- | --- | --- | --- |
| Barr et al. | 2012 | 26.15 | 13.45 | 13 | 31.42 | 13.19 | 12 |
| Fitzgerald et al. | 2008 | 38.8 | 11 | 10 | 53.7 | 8.3 | 10 |
| Huang et al. | 2019 | 53.57 | 5.18 | 30 | 74.4 | 3.49 | 30 |
| Kumar et al. | 2020 | 43.9 | 12.67 | 50 | 50.5 | 14.11 | 50 |
| Li et al. | 2018 | 54.64 | 10.1 | 20 | 62.74 | 11.23 | 21 |
| Lin et al. | 2018 | 23.4 | 3.9 | 30 | 20.3 | 3.5 | 30 |
| Liu et al. | 2017 | 46.42 | 7 | 20 | 56.1 | 7.48 | 20 |
| prikryl et al. | 2013 | 39.22 | 21.11 | 23 | 53.53 | 18.6 | 17 |
| Quan et al. | 2015 | 49.15 | 12.79 | 78 | 54.85 | 14.99 | 39 |
| Singh et al. | 2020 | 35 | 0.97 | 15 | 51.73 | 19.56 | 15 |
