# Supplementary material for: The effects of high-frequency repetitive transcranial magnetic stimulation on negative symptoms in schizophrenia patients: A systemic review and meta-analysis
Source: PLoS One. 2025 Dec 11;20(12):e0337847. doi: 10.1371/journal.pone.0337847 (PMC12697975; doi:10.1371/journal.pone.0337847)
Supplement: Attachment — Submitted filename: Response to reviewers.doc [file pone.0337847.s004.doc]

**Response to reviewers**

We thank the reviewers for their insightful comments, and we are pleased that our manuscript received such positive reviews. Reviewers found, however, several points that need further clarification. We have changed our manuscript according to the reviewers’ valid suggestions and please find below our point-by-point response to each reviewer.

**Reviewer #1:**

1. Objective Section: What specific aspect of rTMS research is considered limited?

**Response:** We thank the reviewer for the valuable suggestion. We have expanded the "Objective Section" to include a discussion on the limitations of rTMS research, particularly focusing on the variability in treatment parameters and the need for standardized protocols.

2. Abbreviations Section: Should the format of upper and lower case letters be standardized?

**Response**: We have standardized the format of abbreviations throughout the manuscript, ensuring consistency in the use of upper and lower case letters as per your suggestion.

3. Introduction Section, Paragraph 3, Sentence 4: There is no mention of comorbid depression in schizophrenia. Why is the significance of rTMS in treating depression specifically emphasized?

**Response**: We thank the reviewer for the valuable suggestion. We have added a discussion on comorbid depression in schizophrenia to highlight its significance and the potential benefits of rTMS in treating both conditions.

4. Should full terms be provided for technical abbreviations when they first appear in the text?

**Response**: We thank the reviewer for the valuable suggestion. We have provided full terms for technical abbreviations when they first appear in the text, as recommended.

5. Should the use of capitalization for technical terms be standardized throughout the manuscript?

**Response**: We thank the reviewer for the valuable suggestion. We have reviewed and standardized the capitalization of technical terms throughout the manuscript. The term about “rTMS” is the standardized form which can be seen in this research 10.1001/jamapsychiatry.2024.0026.

6. Literature Search Section, Sentence 4: Why is the registration number (CRD42023450243) included after “the study selection process”?

**Response**: We thank the reviewer for the valuable suggestion. We originally aim to enhance the transparency and traceability of our research. Now we will delete the registration number (CRD42023450243) in the "Literature Search Section" of the revised manuscript.

7. Subgroup Analysis Section, Paragraph 4: What specific locations are being referred to in terms of differences in “the location of the stimulation”?

**Response**: We thank the reviewer for the valuable suggestion. In the "Subgroup Analysis Section," we only provide a explanation of what is meant by "the location of the stimulation" and discuss how dorsolateral prefrontal cortex (DLPFC) locations may affect treatment outcomes. Because the studies that used PANSS as the primary outcome did not all use the DLPFC as the stimulation site, we wanted to explore whether this site would have an effect on the results.

8. Discussion Section, Paragraph 1, Sentence 4: What specific effect is indicated to be stronger in studies using SANS scores compared to those using PANSS scores?

**Response**: We thank the reviewer for the valuable suggestion. We have now clarified the specific effects observed in studies using SANS scores compared to those using PANSS scores. We discuss how the choice of scale can influence the assessment of negative symptoms and the perceived efficacy of rTMS treatment. This comparison highlights the importance of considering the sensitivity and specificity of different assessment tools when interpreting the effects of rTMS on negative symptoms in schizophrenia patients.

9. Discussion Section, Paragraph 1, Sentence 5: What is the specific relationship between differences in baseline data, frequencies, and stimulation locations and the PANSS and SANS scales?

**Response**: We thank the reviewer for the valuable suggestion. We regret that we were unable to perform a definitive statistical analysis and will continue to investigate solutions in the future. Because we hypothesized that this discrepancy may have occurred as a result of the baseline data from the included studies, more research is needed in the future to explore more appropriate scales.

10. Discussion Section, Paragraph 2, Sentence 2: How can the repeated use of “and” be optimized to improve the logical flow of the sentence?

**Response**: We thank the reviewer for the valuable suggestion. We have rephrased the sentence to improve the logical flow, avoiding repetitive use of "and" and ensuring the sentence reads smoothly.

**Reviewer #2:**

This study reports a systematic review and meta-analysis of rTMS effects on schizophrenia patients, however, a report table presenting the key details for each study, including number of rTMS sessions, stimulation localization in scalp, rTMS stimulation frequency, and mean outcomes for PANSS and SANS (metrics that are discussed in the manuscript) is missing.

Some information currently reported in the Discussion should be moved to the Methods section, specifically details such as the number of studies included in each analysis. Additionally, there is a general lack of clarity regarding the statistical methods applied and the number of studies included in each analysis.

The primary outcome of this study suggests that rTMS improves the negative symptoms of schizophrenia. However, this finding is neither novel nor uniquely revealed by this review and meta-analysis. This information does not add substantial value to the field. Instead, what may contribute more meaningfully is the subgroup comparison conducted subsequently. Again, there is limited clarity on the studies included, their number, and the statistical methods used here. The potentially interesting aspect of this study could lie in the comparison of study characteristics, but the reported diagrams do not compare same indexes of different stimulation characteristics.

There is no description of the statistical analysis between frequencies, no reporting of frequencies per article, no statistical report on differences between session numbers, and no rationale provided for the inclusion criteria for requiring studies with 10 or more rTMS sessions.

Overall, there is a potential impact in the topic and results, however this study does not appear to analyze comparisons that could offer unique insights into the scientific literature—data that could only be derived from a meta-analysis. The study needs to more rigorously present the characteristics of each study and provide a statistically sound comparison of characteristics such as stimulation frequency, number of rTMS sessions, and stimulation location.

**Response**: We thank the reviewer for the valuable suggestion.

(1) We recognize the importance of including a comprehensive reporting form that details the key aspects of each study analyzed. Among our Table 1, we list the number of transcranial magnetic stimulations, stimulation localization, and frequency of transcranial magnetic stimulation, and our supplemental file will also list the mean PANSS and SANS results for each study in the meta-analysis.

(2) We have moved the details regarding the number of studies included in each analysis from the Discussion section to the Methods section to enhance clarity and ensure that all methodological aspects are transparently reported.

(3) To address the lack of clarity regarding the statistical methods applied, we have provided a more detailed description of the statistical tests used and the number of studies included in each analysis in the Methods section.

(4) We understand the need to provide a more nuanced comparison of study characteristics. In our manuscript, we made detailed comparisons of study characteristics and ensured that the reported graphs compared indices for different stimulus characteristics. Based on the subgroup analyses we did, we found that the number of treatments, the frequency of the stimulation, and the location of the stimulation all produced significant differences. However, when we wanted to look for comparative differences between 10 Hz and 20 Hz in high-frequency stimulation, we found that although the effect size of the 20 Hz treatment (-0.40 [-0.67, -0.13]) was greater than that of the treatment of 10 Hz (-0.18 [-0.37, 0.01]), there was an overlap of the two confidence intervals, suggesting that the difference between the two groups may not be statistically significant.

(5) We have revised the inclusive criterion for including studies with 10 or more rTMS sessions.

(6) We have expanded the Discussion section to highlight how our study provides unique insights into the scientific literature, which could only be derived from a meta-analysis. We emphasize the importance of standardized rTMS treatment protocols and consistent outcome measures in future research.

We believe these revisions have significantly strengthened our manuscript and have addressed the concerns raised by your review. We are grateful for the opportunity to improve our work based on your valuable feedback and hope that the revised manuscript meets the standards for publication in your esteemed journal.

Sincerely,

Boxing Wang, Xinyue Zhu, Ling Chen, Liu Shuyun, Chengshi Wang
