# Supplementary material for: The effects of high-frequency repetitive transcranial magnetic stimulation on negative symptoms in schizophrenia patients: A systemic review and meta-analysis
Source: PLoS One. 2025 Dec 11;20(12):e0337847. doi: 10.1371/journal.pone.0337847 (PMC12697975; doi:10.1371/journal.pone.0337847)
Supplement: Attachment — Submitted filename: Response to reviewer.docx [file pone.0337847.s005.docx]

We thank the reviewers for their insightful comments. We are encouraged by the overall positive evaluation and have revised the manuscript in response to the thoughtful suggestions provided. Below, we provide a detailed point-by-point response to Reviewer #2's comments.

**Reviewer #2:**

Reviewer#2: Thank you for your kind response to my previous comments. I appreciate the improved clarity in the description of the statistical procedures. However, I noticed that Table 1 appears to be missing from the submission; I could not locate it within the manuscript, and it is not referenced in the figure legends either. Below, I offer additional comments and suggestions that may help further enhance the manuscript:

Abstract:

• In the opening sentence, please clarify whether the statement "The research on high-frequency repetitive transcranial magnetic stimulation(rTMS)is very little. its effectiveness has hardly been provided conclusive evidence" refers specifically to schizophrenia. Over 700 papers have been published in the past five years on high-frequency TMS in general, so rephrasing may be necessary to avoid misrepresentation.

• Since rTMS is abbreviated in the first sentence, please ensure consistent use of the abbreviation throughout the abstract(e.g.second sentence in the abstract and Practitioner Points section).

• Please write out the full form for RTC before using the abbreviation in the abstract.

**Response:** We thank the reviewer for the valuable suggestion. We clarified the first sentence to emphasize that the focus of the research on repetitive high-frequency transcranial magnetic stimulation (rTMS) is on standardized treatment procedures. We also ensured that the acronym rTMS was used consistently throughout the abstract and expanded the full title before its first use.

Practitioner Points:

• Consider rephrasing the third practitioner point to improve clarity.

**Response:** We thank the reviewer for the valuable suggestion. The third practitioner point has been rephrased to improve clarity.

“High-frequency repetitive transcranial magnetic stimulation may benefit patients with schizophrenia who have significant negative symptoms as well as positive and depressive symptoms.”

• Stimulation Parameters and Table Reference:

• It would be helpful to specify which figure is referred to as Table 1, particularly regarding the report on TMS number of pulses, stimulation location, and frequency. Currently, Figure 1 is the flowchart, and other figures do not indicate stimulation parameters by paper.

• As the systematic review and meta-analysis of stimulation frequency, brain location, and session count are central to your manuscript (as outlined in the introduction), please clearly indicate this information for each study, similar to how the PANSS results are detailed in Supplementary Table S2.

• Table 1 is cited multiple times throughout the manuscript but appears to be missing. If you are referring to the table in one of the PRISMA 2020 Checklist, would be helpful to have an independent table, titled Table 1, with the stimulation parameters per study.

• Please include a legend for Table 1 under the Figure Legends section(or consider renaming the section to" Table and Figure Legends").

**Response:** We thank the reviewer for the valuable suggestion. We have clarified which figure corresponds to Table 1, especially regarding TMS pulse count, stimulation location, and frequency. We have included a detailed table with stimulation parameters for each study; this table is referenced throughout the manuscript. We have added a legend for Table 1 under the "Figure Legends" section, which we have renamed to "Table and Figure Legends" for clarity. The charts are added as images in the section above the manuscript references, while the charts in word form are uploaded as a separate file.

• Search Strategy:

• On inclusion criteria number 2,could you clarify what is meant by "non-invasive stimulation"? Are there additional types of stimulation reported in the studies besides TMS?

**Response:** We thank the reviewer for the valuable suggestion. We have clarified the term "non-invasive stimulation" in the inclusion criteria.

• Data Extraction:

• Please include the stimulation site as part of the extracted data, especially since your findings emphasize the DLPFC. Without this data point, claims regarding the importance of stimulation site may be unsupported.

**Response:** We thank the reviewer for the valuable suggestion. We have incorporated the stimulation site into our extracted data, as suggested. This information is now clearly included in Table 1, which details the stimulation parameters for all studies analyzed. We believe this addition strengthens our findings regarding the importance of the DLPFC stimulation site.

• Outcome Measures:

• In the penultimate sentence, the phrase "addition effective" may have been intended to read "additional effects." If so, please revise for clarity.

**Response:** We thank the reviewer for the valuable suggestion. We have revised the penultimate sentence to clarify that "additional effects" may have been intended to read "additional effects" instead of "addition effective."

• Figures:

• In Figure 1,if the manuscript compares rTMS and sham stimulation groups, consider using the term "sham" in the figure for consistency(or vice versa—use "control" in both the figure and text).If control groups include more than sham stimulation, please clarify this.

• For Figure 1 and other figures, it would be helpful if the legends also describe the table components within the figures, not just the forest plots.

**Response:** We thank the reviewer for the valuable suggestion. We have used the term "control" consistently for the sham stimulation groups. We have updated the legends for Figure 1 and other figures to include descriptions of all table components within the figures, not just the forest plots as the comment. This should provide clearer context and improve the overall understanding of the figures.

Figures 4a-d:

• These figures appear to present data that overlap with Figure 1.To substantiate the claim that 15 sessions of high-frequency rTMS result in greater improvements in negative symptoms, a direct comparison between studies with≥15 sessions vs.<15 sessions is necessary, using only active stimulation groups. The same applies to frequency comparisons (e.g.,20 Hz vs.10 Hz or<20 Hz). Even if these comparisons do not yield statistically significant differences, they remain informative and should be reported in the results section.

• Similarly, to support the claim that DLPFC stimulation is particularly effective, a comparison with other stimulation sites (e.g. ,DLPFC vs. non-DLPFC)would be beneficial.

• Additionally, comparing outcomes like SANS vs. PANSS-positive and CDSS scores could further underscore the specificity of effects on negative symptoms in schizophrenia. You briefly mention the lack of significant effects on these outcomes in the discussion, but including this analysis in the results could strengthen your conclusions.

**Response:** We thank the reviewer for the valuable suggestion. We would have liked to make such a comparison, but only Xiu et al. included experimental groups at 10 Hz and 20 Hz, and we were unable to compare the results of the other articles due to the different baselines. We would prefer that the future studies be able to perform group experiments with different locations, frequencies, and numbers. Therefore, we hope that future studies will be able to perform group experiments with different locations, frequencies, and numbers to aid in the investigation of the efficacy of rTMS. Regarding PANSS-positive and CDSS scores, among the results, we did not find that patients with positive symptoms and depression could benefit from the treatment course of rTMS, which may be further emphasized by the specific efficacy of rTMS on negative symptoms, however, our treatment group was not very abundant, so such results need to be proven by further studies.

We do not emphasize the specific effect of rTMS on negative symptoms, but are more concerned with its broad therapeutic effect, because most schizophrenic patients tend to have a variety of symptom presentations, so we did not compare the results of SANS, PANSS-positive, and CDSS scores.

• Supplementary Materials:

• An additional Supplementary Table(e.g.,S3)detailing stimulation parameters such as intensity, and sham/control procedures could enhance the manuscript’s transparency and utility.

**Response:** We thank the reviewer for the valuable suggestion. To improve the transparency and usefulness of the manuscript, we read the included articles in detail, summarized the treatment procedures in the control group, and added appropriate explanations in the discussion. “In the articles we included, the procedures were consistent with the experimental group except for the orientation of the coils or the dummy coils.”

• References:

• In the discussion section, the manuscript cites Garg et al.as reference 44, however, reference 44 currently corresponds to a different paper (Slotema et al.). It appears that Garg et al. may be missing from the reference list. Please review and correct the reference numbering and ensure all citations are included.

In summary, the manuscript presents a valuable and timely synthesis of high frequency rTMS effects on schizophrenia symptoms. Addressing the points above, especially those related to completeness of results—will substantially strengthen the overall impact and scientific rigor of the paper.

**Response:** Sorry for the careless mistake. We have reviewed and corrected the reference accordingly, we have ensured all citations are included and the reference list is complete
