# Supplementary material for: The effects of high-frequency repetitive transcranial magnetic stimulation on negative symptoms in schizophrenia patients: A systemic review and meta-analysis
Source: PLoS One. 2025 Dec 11;20(12):e0337847. doi: 10.1371/journal.pone.0337847 (PMC12697975; doi:10.1371/journal.pone.0337847)
Supplement: Attachment — Submitted filename: editor.docx [file pone.0337847.s006.docx]

We thank the Editor for their insightful comments, and we are pleased that our manuscript received such positive reviews. Editor found, however, several points that need further clarification. We have changed our manuscript according to the editor’ valid suggestions and please find below our point-by-point response to editor.

**Editor:**

1. Table 1:
• For studies where drug dosage or other parameters were not reported, please replace slashes (“/”) with “not reported” to avoid ambiguity.
• Standardize the description of stimulation targets (e.g., clarify whether “PFC” in Fitzgerald et al., 2008 should be specified as DLPFC or another subregion). For Garg et al. (2016), please explain in the legend what “5, 6, 7” refers to.
• Correct the typo “chlormazapine equivalents” to chlorpromazine equivalents.
• For readability, consider formatting the table to separate participant characteristics (age, sample size, medication) from stimulation parameters (site, frequency, sessions).

**Response:** Thank you for your valuable feedback. We have completed the revisions as requested and have split the original Table 1 into Table 1 and Table 2.

2. Several subgroup analyses (20 Hz vs. 10 Hz, >15 vs. ≤15 sessions, DLPFC vs. other sites) are presented as post-hoc interpretations of effect sizes rather than direct statistical comparisons. This weakens the claim of “protocol-specific superiority.” Please reframe these findings as suggestive rather than conclusive.

**Response:** Thank you for your valuable feedback. We have recognized that our previous wording lacked logical rigor and have revised the text accordingly to ensure no ambiguity arises.

4. Some methodological details (number of studies per analysis, handling of missing data, stimulation site in extracted data) were only clarified after repeated requests. Please ensure that all such details are presented clearly and consistently in the final manuscript, in line with PRISMA and PLOS ONE standards.

**Response:** We carefully verified and ensured that all such details were clearly presented in the final manuscript and complied with PRISMA and PLOS ONE standards.

We appreciate the reviewer's insightful observation.

5. The main conclusions currently recommend 20 Hz, DLPFC stimulation, and >15 sessions. Without direct statistical comparisons, these recommendations risk overstating the strength of evidence. Please adjust the language to emphasize that these findings are preliminary and hypothesis-generating rather than definitive.

**Response:** We appreciate the reviewer's insightful observation. We have adjusted the wording in the text to avoid ambiguity.

We appreciate your valuable feedback and are grateful for the opportunity to improve our manuscript. Please let us know if there are any further concerns or if additional revisions are needed.

Sincerely,

Boxing Wang, Xinyue Zhu, Ling Chen, Liu Shuyun, Chengshi Wang
